# Comparison of uremic toxin removal between expanded hemodialysis and high volume online hemodiafiltrations in different modes

**DOI:** 10.3389/abp.2024.13715

**Published:** 2024-11-29

**Authors:** Jan Biedunkiewicz, Agnieszka Zakrzewska, Sylwia Małgorzewicz, Michał Komorniczak, Katarzyna Jasiulewicz, Natalia Płonka, Agnieszka Tarasewicz, Magdalena Jankowska, Bogdan Biedunkiewicz, Alicja Dębska‐Ślizień, Leszek Tylicki

**Affiliations:** ^1^ Department of Anesthesiology and Intensive Therapy, Medical University of Gdansk, Gdańsk, Poland; ^2^ Department of Nephrology, Transplantology and Internal Diseases, Medical University of Gdansk, Gdańsk, Poland; ^3^ Department of Clinical Nutrition, Medical University of Gdansk, Gdańsk, Poland; ^4^ Medical University of Gdansk, Gdańsk, Poland

**Keywords:** dialysis, hemodialysis, uremic toxins, extended hemodialysis, protein-bound uremic toxins

## Abstract

Various high-efficiency hemodialysis techniques exist, including different online high- volume hemodiafiltration (HDF) modes and expanded hemodialysis (HDx) utilizing dialyzers with medium cut-off (MCO) membranes. This study aimed to evaluate the efficacy of uremic toxin removal among four modalities: (I) HDx, (II) pre-dilution HDF (PRE-HDF), (III) mixed-dilution HDF (MIX-HDF), and (IV) post-dilution HDF (POST-HDF), each applied for 1 week in a randomized order. This research was a single-center, prospective, open-label, exploratory crossover study. The reduction ratio (RR) for small molecular toxins (urea and phosphate), a middle molecular toxin (beta-2-microglobulin, β2M), a large-middle molecular toxin (Chitinase-3-like protein 1, YKL-40), and a protein- bound uremic toxin (indoxyl sulfate, IS) was evaluated during a single mid-week dialysis session. Twelve patients were included, with an average age of 52.5 ± 15.47 years and an average dialysis duration of 42.05 ± 31.04 months. The dialysis parameters, including; post-dialysis weight, session duration, dialysate composition, blood and dialysate flow; rates, dialysate temperature, and anticoagulation dosage, were maintained consistently across all modalities. No significant differences in RR for urea, phosphate, β2M, YKL-40, and IS were observed between the treatments. Although the highest IS clearance, though not statistically significant, was observed with POST-HDF and HDx, the differences were not substantial enough to favor any particular modality as the most effective.

## Graphical Abstract

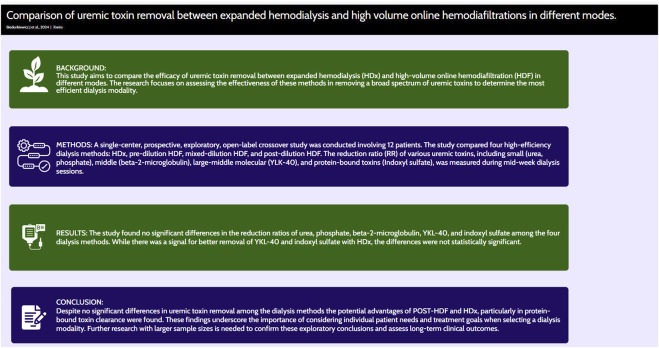

## Introduction

Hemodialysis has seen substantial advancements, evolving from low-flux to high-flux membranes, which are now standard in chronic dialysis therapies (Palmer et al., 2012; Himmelfarb et al., 2020). These technological improvements have led to the development of advanced dialysis methods, such as hemodiafiltration (HDF) in pre-dilution (PRE-HDF), post-dilution (POST-HDF), and mixed-dilution (MIX-HDF) modes, as well as expanded hemodialysis (HDx) employing MCO membranes. These new methods have enhanced clearance profiles, significantly improving the removal of a wider range of uremic toxins, especially larger middle molecules, which traditional high-flux dialysis failed to eliminate effectively (Pedreros-Rosales et al., 2023; Zhang et al., 2022). The CONVINCE trial demonstrated that high-volume POST- HDF, delivering at least 23 Liters per session, was associated with a reduced risk of all-cause mortality compared to conventional high-flux hemodialysis, especially among older, non-diabetic patients with arteriovenous fistulas and no history of cardiovascular disease (Blankestijn et al., 2023). In Asian countries, PRE-HDF has also shown a potential for improved survival rates over conventional high-flux hemodialysis. A Japanese cohort study involving 5,000 patient pairs treated with either standard hemodialysis or pre-dilution HDF indicated that PRE-HDF might offer better overall and cardiovascular survival, particularly with high substitution volumes (>40.0 L per session) (Kikuchi et al., 2019).

Smaller studies have suggested HDx might improve patient quality of life and reduce symptoms such as restless leg syndrome and pruritus (Zhang et al., 2022). However, a randomized controlled trial by Lee et al. found no significant cardiovascular differences between HDx and POST-HDF (Lee et al., 2021). HDx remains technically simple, similar to conventional hemodialysis, which makes it increasingly popular, especially in patients with high comorbidity, long dialysis duration, or who are not candidates for kidney transplantation. Although numerous studies have compared different HDF techniques or HDx with individual HDF modes, comprehensive comparisons among all high-efficiency dialysis methods are scarce (Mitchell et al., 2023; Zakrzewska et al., 2024). This study aimed to evaluate the efficacy of all available high-efficiency dialysis techniques at our center in terms of solute clearance across a broad spectrum of uremic toxins.

## Materials and methods

### Study design

This study was a single-center, prospective, exploratory, open-label, crossover trial. The objective was to compare the removal of uremic toxins across four high-efficiency dialysis methods: (I) HDx, (II) online pre-dilution HDF (PRE-HDF), (III) online mixed-dilution HDF (MIX-HDF), and (IV) online post-dilution HDF (POST-HDF). Patients underwent each modality for a week in a randomized sequence. The effectiveness of toxin removal was determined by calculating the reduction ratio (RR) for small molecular toxins such as urea (MW 60 Da) and phosphate (MW 95 Da); a middle molecular toxin, beta-2-microglobulin (β2M, MW 11,800 Da); a large middle molecular toxin, Chitinase-3-like protein 1 (YKL-40, MW 40,000 Da); and a protein-bound uremic toxin (PBUT), indoxyl sulfate (IS, MW 213 Da). Toxin reduction was evaluated during a single mid-week dialysis session. The study followed the Declaration of Helsinki’s guidelines and was approved by the Medical University of Gdansk’s Ethical Committee (NKBBN/479-759/2022; 18 November 2022).

### Patients

Participants included adult patients with end-stage renal disease (ESRD) who had been receiving standard high-flux hemodialysis or online HDF (either pre- or postdilution) three times per week for a minimum of 6 months. Eligible patients had a single-pool Kt/V for urea (spKt/Vurea) greater than 1.2, weighed between 60 and 89 kg, and had a dialysis blood flow of at least 350 mL/min through a fistula or arteriovenous catheter. Exclusion criteria included single-needle dialysis, the use of temporary non-tunneled catheters, poor compliance with dialysis procedures, hemodynamic instability during dialysis sessions, life expectancy of less than 6 months, hospitalization within the last 30 days, active inflammation or cancer, liver cirrhosis, and hypoalbuminemia (albumin <30 g/L).

### Dialysis prescription and equipment

All dialysis treatments were performed using the Fresenius 5008 machine with the AutoSub Plus system (Fresenius Medical Care, Bad Homburg, Germany). Online HDF sessions were carried out with FX 100 high-flux dialyzers (surface area: 2.2 m^2^; UF coefficient: 73 mL/h × mmHg; Fresenius Medical Care). HDx treatments utilized Terranova 400 MCO dialyzers (surface area: 1.7 m^2^; UF coefficient: 48 mL/h × mmHg; Baxter, Alliston, ON, Canada). Each dialysis session lasted 4 h, with a dialysate temperature of 36.5°C. Blood flow and dialysate flow rates were set to 350 and 500 mL/min, respectively. Dry weight was verified using bioimpedance spectroscopy prior to the study. Ultrafiltration for each session was adjusted based on the patient’s interdialytic weight gain and fluid intake, along with bloodline priming volume. Neither ultrafiltration nor sodium profiling was employed. The dialysate composition was: Na 138–140 mmol/L; K 2.0–3.0 mmol/L; HCO₃ 32 mmol/L; Ca 1.25–1.5 mmol/L; Mg 0.5 mmol/L; Cl 110 mmol/L; glucose 1.0 g/L. Most patients (83.3%) used a potassium concentration of 2.0 mmol/L, and 91.7% used a calcium concentration of 1.25 mmol/L. Heparin was administered as a bolus and a continuous infusion according to current practices. Sterile, non-pyrogenic substitution fluid was generated online via ultrapure dialysate filtration. The AutoSub Plus system automatically adjusted the substitution and convection rates based on pressure pulse attenuation and transmembrane pressure, optimizing ultrafiltration while preventing excessive hemoconcentration. Dialysis settings, such as post-dialysis weight, session length, dialysate composition, and anticoagulation, were kept consistent across all modalities. Medications remained unchanged throughout the study.

### Monitoring of uremic toxins concentration

Blood samples for assessing toxin RR were collected before and after mid-week dialysis session. Post-dialysis sample collection was done at the dialysis session end from the arterial needle after decreasing the blood flow rate to 50 mL/min for 15s to avoid recirculation. RR of toxins was calculated by the following equation:

$$\text{RR }\left(\%\right)=\left[1-\left(\frac{cCpost}{Cpre}\right)\right]\;x\;100$$

*Cpre* and *Cpost* refer to toxin concentration of pre- and post-dialysis session, respectively while *BWpost* is the body weight at the end of the session. *Cpost* was corrected for hemoconcentration as follows:

$$\text{cCpost}=\frac{cCpost}{\left[1+\left(\frac{BW}{0.2\left(BWpost\right)}\right)\right]}$$

### Laboratory measurements

Urea and phosphate in serum were quantified using routine colometric methods (Abbott GmbH and Co. Wiesbaden, Germany).

Serum beta-2-microglobulin levels were determined with a reagent kit (Abbott GmbH and Co. Wiesbaden, Germany). The Alinity c β2-microglobulin reagent utilizes latex particles coated with IgG antibodies specific to human β2M, and agglutination is measured via a turbidimetric method. The detection limit was 0.110 mg/L, with an inter-assay CV of 4.1% and an intra-assay CV of 4.2%. The measurement range was 0.97–2.64 mg/L.

Serum YKL-40 (CHI3L1) was quantified using the YKL-40 Human Sandwich ELISA Kit (Thermo Fisher Scientific Inc.). This assay involves an anti-human YKL-40 antibody that binds to YKL-40 in the sample, followed by the addition of a biotin-conjugated detection antibody. The sensitivity was 10.83 pg/mL, with an assay range of 78–5,000 pg/mL, an inter-assay CV of 7.2%, and an intra-assay CV of 2.3%. Serum YKL-40 (CHI3L1) was quantified using the YKL-40 Human Sandwich ELISA Kit (Thermo Fisher Scientific Inc.). This assay involves an anti-human YKL-40 antibody that binds to YKL-40 in the sample, followed by the addition of a biotin- conjugated detection antibody. The sensitivity was 10.83 pg/mL, with an assay range of 78–5000 pg/mL, an inter-assay CV of 7.2%, and an intra-assay CV of 2.3%.

Serum indoxyl sulfate (IS) levels were determined using a competitive ELISA kit (Fine Test^1^). In this method, IS in the sample competes with the IS coated on the microtiter plate for the binding sites of a biotinylated detection antibody. The resulting colorimetric reaction was measured at 450 nm. The detection range for the assay was 1.563–100 ng/mL, with an intra- assay precision of 5.1% (low concentration: 3.16 ± 0.16) and inter-assay precision of 5.4%.

### Statistical analysis

Continuous variables were expressed as means with standard deviations (SD), while categorical data were represented as percentages. The Shapiro–Wilk test was used to evaluate the normality of continuous variables. The Wilcoxon signed-rank test or ANOVA was utilized for repeated measures comparisons. Statistical significance was considered at *p* < 0.05. All analyses were performed using Statistica 13.3 (TIBCO Software Inc.; Palo Alto, CA, United States).

## Results

### Characteristics of patients

12 patients met inclusion criteria and were enrolled to the study, 11 men (92%) and 1 woman, in mean age of 52.5 ± 15.47 years. Hypertension was diagnosed in 10 (83%) patients. A description of the study group is presented in Table 1.

**TABLE 1:** Characteristics of the study group.

Gender (Men/Women)	11/1
Causes of ESRD (n/%)	
Autosomal Dominant Polycystic Kidney Disease	4/33
Glomerulonephritis (primary or secondary)	3/25
Hypertensive nephropathy	2/17
Renal malformation	1/8
Interstitial nephropathy	1/8
Other	1/8
Age (years)	52.5 (15.5)
AACI (points)	4.5 (2.2)
Dialysis vintage (months)	42.5 (31.0)
Body Mass Index (kg/m^2^)	23.8 (3.6)
Weight (kg)	73.7 (14.2)
spKt/V urea	1.5 (0.3)
Hemoglobin (g/dL)	10.9 (0.9)
Albumin (g/L)	33.1 (4.9)

ESRD, end-stage renal disease; AACI, Age Adjusted Charlson Comorbidity Index. Data are presents as mean (SD) or number (%).

### Dialysis parameters

Dialysis session time, blood flow rate, and dialysate flow rate were constant during all modalities. All patients achieved the minimum level of convection for high volume online HDF. Mean (standard deviation) total convection for post-HDF, pre-HDF and mix-HDF were 25.6 (3.8), 61.5 (7.2) and 47.1 (11.4) L, respectively. The arterial and venous dialysis pressure did not differ between tested treatments. Detailed delivered dialysis parameters are presented in Table 2. All patients in all sessions have achieved high-volume convection defined as ≥23 L of substitution fluid.

**TABLE 2:** Delivered dialysis parameters during the study treatments.

	PRE-HDF	MIX-HDF	POST-HDF	HDX
Real Time min	240	240	240	240
Blood flow mL/min	350	350	350	350
Dialysate flow mL/min	500	500	500	500
Ultrafiltration L/session (SD)	2.45 (0.8)	2.29 (0.74)	2.19 (0.52)	2.33 (0.62)
Total convection L	61.5 (7.2)	47.1 (11.4)	25.6 (3.8)	NA

Note: Ultrafiltration refers to the fluid removed from the patient during the dialysis session. Total convection is the total volume of fluid moved by convection during the session, which includes both the patient’s dehydration volume and the volume of replacement fluid administered.

### Effectiveness in the removal of toxins

No difference was seen in RR between treatments for small middle and large molecules, neither for the protein bound uremic molecule (Table 3). The best degree of IS clearance, although statistically insignificant, was obtained during POST-HD and HDX.

**TABLE 3:** Molecules reduction ratio, according to size and treatment modality, Data expressed as means (SD).

Molecule	Size (Da)	HDF-pre	HDF-mix	HDF-post	HDx	*P*-value
Urea	60	0.73 (0.1)	0.72 (0.06)	0.75 (0.07)	0.72 (0.06)	0.67
Phosphate	95	0.54 (0.16)	0.56 (0.08)	0.59 (0.11)	0.56 (0.12)	0.86
Beta-2-microglobulin	11.000	0.62 (0.15)	0.69 (0.13)	0.7 (0.1)	0.67 (0.06)	0.41
YKL-40	40.000	0.31 (0.12)	0.32 (0.12)	0.32 (0.14)	0.36 (0.19)	0.92
Indoxyl sulfate	213	0.36 (0.23)	0.40 (0.17)	0.40 (0.17)	0.49 (0.17)	0.51

**FIGURE 1:**
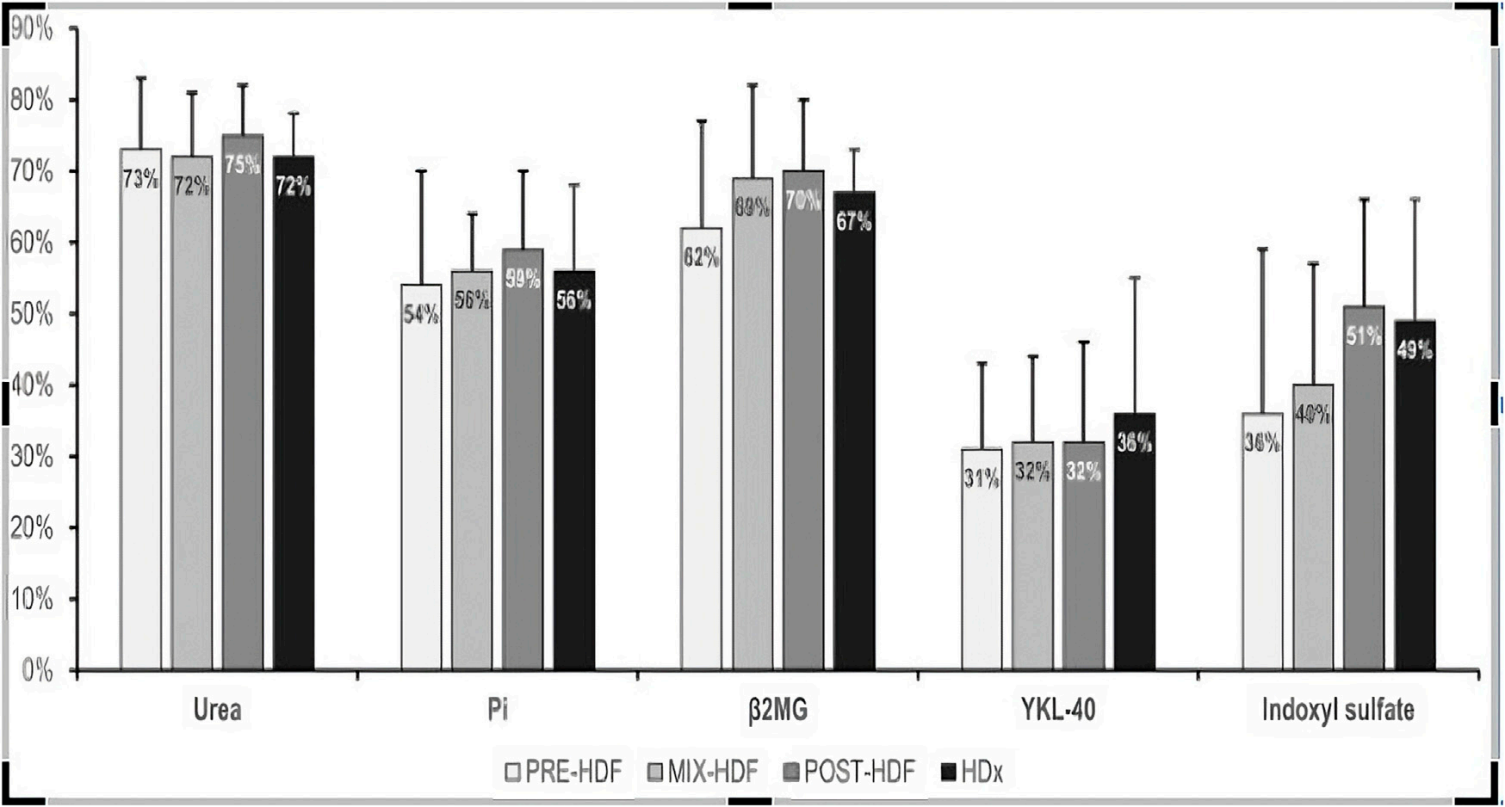
Mean reduction ratio of uremic toxins during the study treatments.

The impact of studied blood purification modalities on the removal of the toxins is displayed on Figure 1.

**FIGURE 2:**
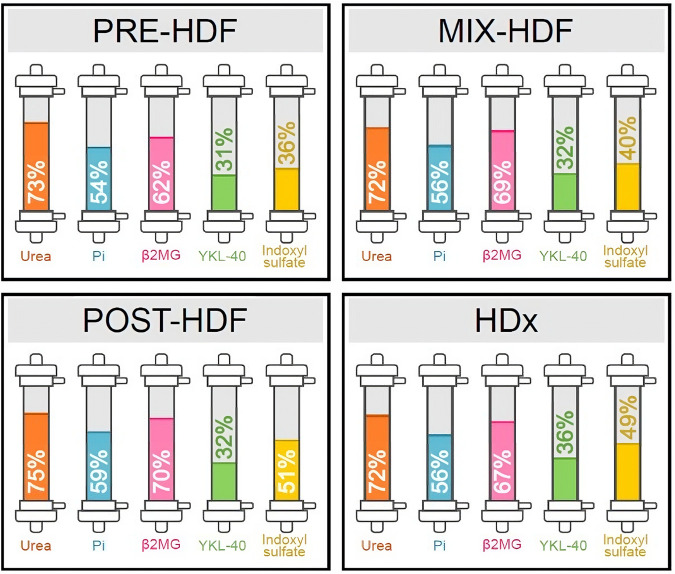
Comparative efficacy profiles of dialysis methods against specific toxins. This figure illustrates mean reduction ratio of uremic toxins during the study treatments.

Figure 2 compares each treatment modality’s capacity to remove a whole profile of molecules. Again, there was no difference between modalities.

## Discussion

The main purpose of all blood purification techniques is to remove uremic toxins from the patient’s blood. Our study showed no significant differences in uremic toxin removal between the compared methods of high-efficiency dialysis. Ensuring high-volume convection, i.e., 23 Liters (POST-HDF), 60 Liters (PRE) and 45 Liters (MIX-HDF) on average, these methods do not differ from each other in the clearance efficiency, nor do they differ from hemodialysis using MCO membranes.

We analyzed a broad spectrum of uremic toxins, including small-molecule toxins (urea), medium-molecule toxins (β2M), large medium-molecule toxins (YKL-40), and toxins bound to and proteins (IS). The proposed cross-over design study excluded the influence of patient-related variability on the obtained results and ensured the same technical parameters of dialysis, including its time, blood and dialysis fluid flow rate limited bias in this regard.

The technical development of dialysis mainly resulting from the introduction of high-flux dialyzers, dialyzers with MCO membranes and dialysis methods based on high-volume convection has led to very effective removal of most uremic toxins. This is especially true for small water-soluble compounds with low molecular mass (<500 Da) whose reduction ratio during the dialysis is very high for most commonly used methods. The ability to remove larger uremic toxins relies largely on the convection amount. The high-flux dialyzer, when applied in the standard hemodialysis, has a molecular mass cutoff of 25 kDa, being boosted up to 30 kDa in HDF mode. In our study, we did not show significant differences between studied treatments in the removal of urea, phosphate and β2M. The degree of purification was high, exceeding 70% for urea. Previous studies have shown slightly better clearance rate for small molecules during POST-HDF compared to PRE-HDF, which was explained by the smaller concentration gradient of these toxins across the dialysis membrane in the later mode. These toxins are removed during dialysis primarily by the diffusion mechanism. In some studies, slightly better clearance was observed during POST-HDF compared to PRE-HDF also for medium size toxins (500–25,000 D) such as β2M or alfa-1 microglobulin (Duval-Sabatier et al., 2023; Meert et al., 2008). The purification potential of MIX-HDF has been investigated only in few studies, indicating that the removal of β2M is also slightly better than in PRE-HDF and similar to POST-HDF (Park et al., 2021; de Sequera et al., 2013). In our study, β2M removal during PRE-HDF was the lowest among the tested methods, but the differences did not reach statistical significance. In previous studies, HDF patients in the highest β2M tertile tended to have lower replacement fluid volume than patients in the middle and lowest tertiles (Kanda et al., 2021). This points to an important advantage of our protocol in which all techniques were applied to the same patient. Careful selection of the study group allowed for optimal convection in each applied method. Therefore we were able to control important clinical confounders and our conclusions on comparability between single sessions of all used modalities are more reliable.

Large-middle uremic toxins (25–58 kDa) are important molecules, the accumulation of which is associated with numerous complications of chronic kidney disease and an increased risk of cardiovascular complications. They are poorly removed during hemodialysis using high-flux dialyzers; some but unsatisfactory improvement has been achieved in online HDF methods with high-volume convection (Rosner et al., 2021). The new class of MCO membranes with a large pore radius of 5 nm and high internal convection using in so called extended hemodialysis were intended to provide more efficient clearance for these toxins. Clinical studies in this area to date has yielded inconclusive results. Some studies showed better removal of large molecules involving λ free light chains and YKL-40 as compared to PRE-HDF (Kim et al., 2019), MIX-HDF (Eiamcharoenying et al., 2022), and POST-HDF (Kirsch et al., 2017; Hadad-Arrascue et al., 2022). On the other hand, other studies do not show differences between HDx and POST-HDF in this regard (Garcia-Prieto et al., 2018; Maduell et al., 2019a). Moreover, Maduell et al. showed no differences in removal efficacy between HDx and POST-HDF analyzing the global removal score, taking into account 6 different uremic toxins (Maduell et al., 2019b). In our study, HDx showed slightly better removal of YKL-40 compared to other methods, achieving a clearance rate of 36%, though the differences were not statistically significant.

Protein-bound uremic toxins (PBUTs) like indoxyl sulfate (IS) and p-cresol sulfate are crucial prognostic markers. Elevated serum levels of these toxins are linked to cardiovascular events and contribute to vascular diseases such as arteriosclerosis, endothelial inflammation, oxidative stress, and vascular calcification (Lin et al., 2015; Opdebeeck et al., 2019). Despite their relatively small molecular weight, PBUTs are poorly cleared during conventional hemodialysis, even with high-flux dialyzers. Their removal is hindered by their strong binding to plasma albumin, as only the unbound fraction can pass through the dialysis membrane. In the kidneys, PBUTs are primarily eliminated via tubular secretion (Masereeuw et al., 2014). The efficiency of PBUT removal during hemodialysis may depend on several factors, including dialyzer size, protein loss, dialysis duration, dialysate flow rate, protein adsorption to the dialysis membrane, and the extent of PBUT-albumin dissociation during contact with the membrane (Sanchez-Ospina et al., 2024; Huang et al., 2009). There was initial hope that high-volume HDF and HDx might improve PBUT removal. However, prior studies have produced mixed results regarding whether high- volume HDF is superior to conventional HD (Abad et al., 2016; Panichi et al., 2017; Krieter et al., 2010; Snauwaert et al., 2020; van Gelder et al., 2020), and no substantial evidence has shown that HDx increases PBUT clearance (Deltombe et al., 2015; Ronco et al., 2017). No comparative studies exist that assess PBUT removal across all high-efficiency modalities. In our research, the highest IS clearance was observed with POST-HD and HDx, although the difference was not statistically significant.

The novelty of our study lies in the fact that no previous research has compared the efficacy of all forms of online HDF and HDx in a single analysis. Our study also has several strengths: 1) the crossover design was chosen to mitigate interpatient variability; 2) basic dialysis parameters were standardized across all treatment methods; 3) all HDF modalities were performed at high volumes, which is known to offer the best long-term outcomes; 4) the removal rate was adjusted for hemoconcentration during dialysis. Our findings have practical implications, suggesting that treatment selection should consider the characteristics of uremic toxins. However, we acknowledge the limitations of our study. The study population may not reflect a typical European dialysis cohort, as we only included one woman, potentially affecting the homogeneity of the group, though the crossover design should minimize this issue. Another limitation is the small sample size, which may have prevented some differences from reaching statistical significance. Additionally, we analyzed the reduction ratio during only a single dialysis session. Future research could benefit from assessing the long-term effects of these modalities on toxin concentrations. A valuable complement to the analysis would be a comparison of the treatment effectiveness of the tested high-efficiency methods to the results obtained during standard hemodialysis. The influence of hemoconcentration on the concentration of all toxins and their reduction ration was taken into account in the study. For this purpose, toxin concentrations were corrected taking into account the change in the patient’s weight during dialysis but an important limitation of the analysis is the lack of hematocrit-based hemoconcentration calculations. Considering these limitations, the findings should be viewed as exploratory.

In conclusion, this study found no significant differences in the clearance of a broad range of uremic toxins across four high-efficiency dialysis methods—PRE-HDF, MIX-HDF, POST-HDF, and HDx. While some trends were observed, particularly in PBUT removal, these differences were not substantial enough to recommend any one modality as the most effective.

## Data Availability

The raw data supporting the conclusions of this article will be made available by the authors, without undue reservation.

## Footnotes

1
http://www.fn-test.com
